# Molecular and cellular mechanisms underlying the failure of mitochondrial metabolism drugs in cancer clinical trials

**DOI:** 10.1172/JCI176736

**Published:** 2024-02-01

**Authors:** Karthik Vasan, Navdeep S. Chandel

**Affiliations:** Department of Medicine, Biochemistry and Molecular Genetics, Northwestern University Feinberg School of Medicine, Chicago, Illinois, USA.

The majority of cancer cells have a functional mitochondrial electron transport chain (ETC). Mitochondrial complex I is the primary entry point into the ETC, where oxidative phosphorylation occurs, generating ATP as an energy source for powering the cell. Electrons are transferred through a chain of mitochondrial protein complexes (complex I, II, III, and IV) to the final electron acceptor, molecular oxygen, while protons are pumped by complexes I, III, and IV to create an electrochemical proton gradient, ultimately driving ATP synthesis through complex V. The ETC can function optimally even in hypoxic conditions, allowing solid tumors, which often have limited oxygen availability, to maintain mitochondrial respiration. Mitochondrial ETC function is intrinsically linked to the oxidative tricyclic acid (TCA) cycle, which supports tumor growth by enabling macromolecule biosynthesis (1). Genetic and pharmacologic inhibition of the ETC prevents de novo pyrimidine synthesis and oxidative TCA cycle flux, supporting lipid, heme, aspartate, and asparagine production, all of which act together to decrease primary tumor growth and metastasis (2). Furthermore, mutations in ETC genes are generally selected against across various types of cancer (3). Thus, mitochondrial metabolism is an essential, dynamic process throughout tumorigenesis, with metabolic flexibility serving the tumor’s needs at every stage, from initiation to metastasis. Ultimately, the metabolic demand imposed by driver mutations on specific tissue lineages, coupled with nutrient or metabolite availability of tissue microenvironment, determines the dependency on any intracellular metabolic pathways, including ETC-linked pathways. Owing to the critical role of mitochondria in tumor growth, there is considerable interest in translating inhibition of mitochondrial ETC or TCA cycle metabolism into clinical practice (1). However, clinical trials with drugs targeting mitochondrial metabolism, including metformin, have largely failed (4). Here, we discuss the mechanisms underlying why these trials have failed and a possible path forward to successfully targeting mitochondria metabolism, using metformin as an example, for cancer therapy.

## Toxicity of mitochondrial metabolism–targeting drugs

Multiple inhibitors that target mitochondrial metabolism have been tried in clinical trials, including CPI-613 (also known as devimistat), CB-839 (also known as telaglenastat), ONC201, and IACS-010759. CPI-613 is a lipoate analog that purportedly inhibits 2 tricyclic acid (TCA) cycle enzyme complexes, α-ketoglutarate dehydrogenase and pyruvate dehydrogenase. Although the exact mechanism of CPI-613’s anticancer activity is unclear, promising phase I outcomes in pancreatic cancer and acute myeloid leukemia (AML) trials have been reported (NCT01835041 and NCT01034475). However, CPI-613 in combination with FOLFIRINOX did not have a survival benefit compared with FOLFIRINOX alone for patients with previously untreated metastatic pancreatic ductal adenocarcinoma (AVENGER 500 trial, NCT03504423). CB-839 is a glutaminase inhibitor that prevents glutaminolysis from feeding the TCA cycle. In a phase II clinical trial, CB-839 failed to provide benefit compared with standard-of-care immunotherapy in patients with stage IV non–small cell lung cancer (NSCLC) tumors harboring loss-of-function mutations in KEAP1 (KEAPSAKE trial, NCT04265534). IACS-010759 is a potent mitochondrial complex I inhibitor that showed impressive preclinical efficacy in AML and brain cancer models and was well tolerated in mouse models (5, 6). However, a recent phase I trial in relapsed/refractory AML revealed a narrow therapeutic index and dose-limiting toxicities, such as lactic acidosis and neurotoxicity (5). Targeting mitochondrial metabolism is hindered by toxic side effects limiting efficacious antineoplastic dosing (7). This is not surprising given that mitochondrial electron transport chain (ETC) function is necessary for the function of normal tissue, including conventional T cells (8). Thus, successful clinical translation of targeting mitochondrial metabolism requires an effective, yet safe and well-tolerated, drug. Additionally, two promising phase I studies (NCT03416530 and NCT03134131) demonstrated that ONC201, an activator of the mitochondrial caseinolytic protease P (CLPP) that results in impaired respiratory function, demonstrated efficacy as a monotherapy in H3K27M-mutant diffuse midline gliomas (9). ONC201 was originally developed as a brain-penetrant dopamine receptor D2 (DRD2) antagonist and is tolerable for cancer therapy (10, 11).

One such drug that targets mitochondrial metabolism is metformin, a biguanide widely used as first-line treatment for type II diabetes mellitus. Laboratory-based studies have shown that metformin has an anticancer effect, and retrospective studies have reported a reduced incidence of cancer diagnoses and cancer-related deaths in patients treated with metformin (1). While metformin has various proposed mechanisms of action, inhibition of mitochondrial complex I is required for metformin’s antitumorigenic effect (12, 13). Inhibition of mitochondrial complex I decreases glucose flux into macromolecule biosynthesis by inhibiting the oxidative TCA cycle. Metformin has been shown to have an antitumorigenic effect in patients with ovarian and breast cancer by targeting tumor cell–intrinsic mitochondrial metabolism (14, 15). In patients with breast cancer, integrated pharmacodynamic analysis has identified two metabolic adaptation pathways for metformin resistance: increased glucose flux and increased transcription of oxidative phosphorylation genes. Nevertheless, some clinical trials have reported efficacy of metformin in various cancers, and other trials have not shown robust anticancer efficacy (16). For instance, a recent stage II clinical trial in ovarian cancer demonstrated better-than-expected overall survival in the metformin-treated group, while a phase III randomized trial that included over 3,600 patients with breast cancer failed to show an improvement in disease-free survival with the addition of metformin to standard-of-care treatment (4). Similarly, metformin failed to show any benefit in phase II randomized trials in NSCLC when combined with chemoradiotherapy (17, 18). Although various other studies have shown promising results, the clinical benefit of metformin remains unclear (16). Due to the inconsistency between preclinical data on metformin’s antitumorigenic effects and clinical trial results, a better understanding of the molecular mechanisms required for metformin’s action is needed.

## The cellular environment influences mitochondrial metabolism inhibitors

**Figure 1:**
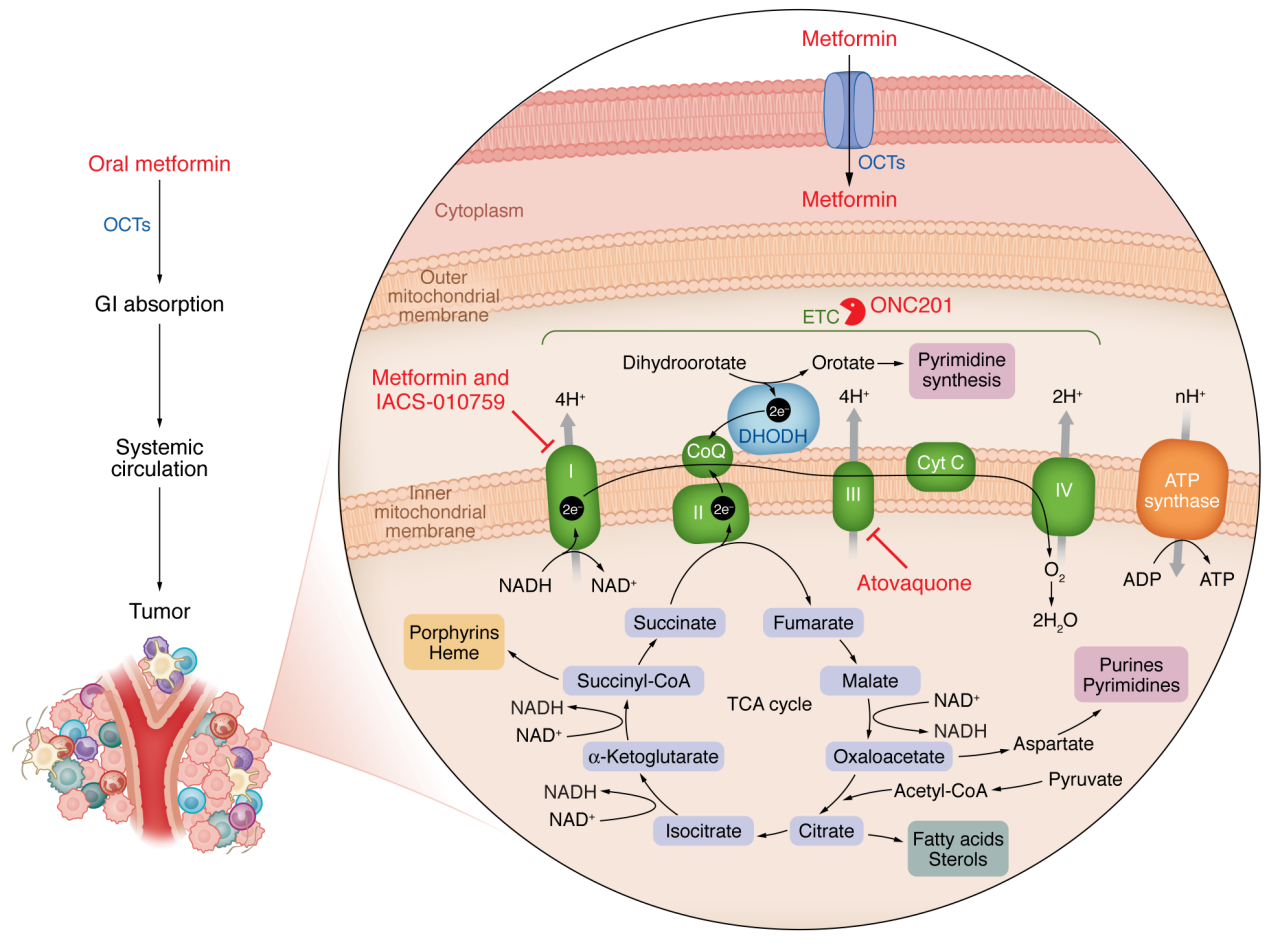
Drugs that target the mitochondrial electron transport chain in cancer cells have antineoplastic properties. Mitochondrial electron transport chain (ETC) is necessary to sustain metabolites required for cancer cell growth. Metformin’s primary anticancer mechanism involves the inhibition of mitochondrial ETC complex I. The drug’s safety and efficacy are associated with organic cation transporters (OCTs), which have varying presence across regular tissues and cancers. Metformin relies on OCTs for cell entry, and variability in OCT expression levels along with the ability of cancer cells to metabolically adjust to the tumor microenvironment might account for inconsistent results in clinical trials. New drugs on the horizon targeting the ETC include the antimalaria drug atovaquone, an inhibitor of mitochondrial complex III, and ONC201, an activator of mitochondrial protease caseinolytic protease P (CLPP) that degrades ETC proteins. The molecular determinants that would make these drugs effective and their specific therapeutic window need to be addressed.

As a mitochondrial ETC inhibitor, metformin should theoretically be toxic, but it has a high safety profile, which is attributed to its reliance on organic cation transporters (OCTs) for cellular entry (Figure 1). OCTs are highly abundant in normal kidney, gut, and liver cells and can transport various compounds, including polyamines, thiamine, carnitine, dopamine, and acetylcholine. When administered orally, metformin has a “flip-flop” pharmacokinetic profile, with slower gut absorption than kidney elimination, resulting in higher gut concentrations (19). The heterogeneity of the tumor response to metformin may be due in part to variability in OCT expression. A metabolism-focused CRISPR-based genomic screen found that OCT3 was required for metformin-induced cell death (1, 20). Expression of OCTs has been shown to be necessary for metformin’s antitumor effect in certain cancers such as squamous cell carcinomas of the head and neck and breast cancer (20). The lack of patient stratification by OCT expression in clinical trials may contribute to the variability in metformin’s efficacy against tumors.

Another factor to consider is the ability that tumor cells possess for obtaining the necessary building blocks for growth from their surrounding environment, allowing them to sequester energy in the absence of the mitochondria and consequently making them resistant to metformin (21). Thus, in preclinical mouse models of cancer, the combination of metformin, which is known to decrease intracellular asparagine, coupled with asparaginase to decrease serum asparagine levels, diminishes tumor growth (22).

Tumor hypoxia, a widely acknowledged obstacle to radiation, chemotherapy, and immunotherapy treatments, diminishes the effectiveness of immune checkpoint inhibitors such as anti-PD1 due to its immunosuppressive nature (23). Although mitochondrial ETC is necessary for T cell function, it is unclear whether any of the mitochondrial ETC inhibitors in clinical trials accumulate in T cells or myeloid cells to the levels needed for decreased respiratory function or altered immune function. Interestingly, targeting metformin specifically to myeloid cells using nanoparticles can improve anti-PD1 therapy in mouse models of cancer (24). Additionally, the severity of tumor hypoxia positively correlates with the cancer’s metastatic potential. As a result, there is growing interest in employing mitochondrial ETC inhibitors to enhance tissue oxygen levels (25). Atovaquone is promising drug on the horizon; it is already clinically administered alongside proguanil to prevent and treat *Plasmodium falciparum* malaria. Atovaquone acts as a competitive inhibitor of ubiquinol, leading to specific inhibition of the ETC complex III in *Plasmodium falciparum* at an IC_50_ value of 2 nM. In contrast, its IC_50_ value for human mitochondrial complex III is 460 nM, suggesting low concentrations are safe yet effective. Notably, this compound has the capacity to decrease both oxygen consumption and cell proliferation in various cancer cell lines. A recent phase I clinical study confirmed the potential of atovaquone in mitigating tumor hypoxia in NSCLC (NCT02628080). This trial employed noninvasive PET-CT imaging, with [18F]-fluoromisonidazole or [18F]-fluoroazomycin arabinoside tracers (26). These tracers undergo irreversible intracellular enzymatic reduction in low-oxygen conditions, allowing for the assessment of tumor hypoxia. It will be important to elucidate the mechanisms that determine the preferential accumulation of atovaquone in tumors or specific normal tissues to better comprehend its therapeutic potential and limitations.

## Metabolic therapy as a targeted treatment

Moving forward, it may be appropriate for metabolic therapy in clinical settings to follow the example of targeted therapy, which is the cornerstone of precision medicine. Targeted therapy is a type of cancer treatment that targets specific proteins critical for cell growth, proliferation, and metastasis. Imatinib, a small molecule that targets the BCR-ABL fusion protein commonly found in patients with chronic myelogenous leukemia, and Herceptin, a monoclonal antibody that targets the Her2/neu receptor expressed in certain types of breast cancer, are classic examples of targeted therapy. Only patients who express the corresponding target protein are considered for treatment with these drugs. Similarly, identifying tumors that express OCTs can indicate patients who are good candidates for metformin therapy. Genetic mutations and cancer drivers can inform the use of metformin. For example, tumors deficient in homologous recombination, such as those with BRCA mutations, rely on mitochondrial metabolism to regenerate ATP for PARP-dependent repair mechanisms, making them vulnerable to inhibitors such as metformin. Furthermore, a variety of human tumors tend to demonstrate high TCA cycle flux, which correlates with metastatic potential as well as tumors that become resistant to standard-of-care therapy (27). Thus, these tumors could be suitable to ETC inhibition by metformin, provided they have robust expression of OCTs. Moreover, the use of PET imaging to assess hypoxia as well as other techniques to assess in vivo tumor metabolism (28) could be integrated as part of future clinical trials to assess efficacy of drugs that target ETC. By understanding how genetics drive metabolic vulnerabilities to mitochondrial inhibitors such as metformin through expression of OCTs, deciphering the best combination therapy regiments, and assessing patient tumor metabolic status, we can stratify patients for effective, evidence-based targeted metabolic therapy.

## Version 1 02/01/2024

Electronic publication
